# The relationship between the activates of antioxidant enzymes in red blood cells and body mass index in Iranian type 2 diabetes and healthy subjects

**DOI:** 10.1186/2251-6581-11-3

**Published:** 2012-08-02

**Authors:** Ehsaneh Taheri, Mahmoud Djalali, Ahmad Saedisomeolia, Ali Malekshahi Moghadam, Abolghasem Djazayeri, Mostafa Qorbani

**Affiliations:** 1 Department of Nutrition and Biochemistry, School of Public Health, Tehran University of Medical Sciences, Tehran, Iran; 2 Faculty of Veterinary Medicine, University of Tehran, Tehran, Iran; 3 Department of Epidemiology & Biostatistics, School of Public Health, Tehran University of Medical Sciences, Tehran, Iran; 4 Endocrinology and Metabolism Research Center, Tehran University of Medical Sciences, Tehran, Iran

**Keywords:** Diabetes mellitus, Antioxidant enzymes, Total antioxidant capacity

## Abstract

**Background:**

Diabetes mellitus is a metabolic disorder characterized by increased production of free radicals and oxidative stress. The aim of this study was to evaluate the activity of antioxidant enzymes, superoxide dismutase (SOD), glutathione reductase (GR), and glutathione peroxide (GSH-PX) in type 2 diabetic patients compared with healthy subjects.

**Methods:**

This cross-sectional study was conducted on 100 type 2 diabetic patients and 100 healthy controls. Total antioxidant capacity and fasting serum levels of SOD, GR, and GSH-Px were measured. All data were analyzed using SPSS software compatible with Microsoft Windows.

**Results:**

The activity levels of SOD were lower in diabetic patients (111.93 ± 354.99 U/g Hb) than in healthy controls (1158.53 ± 381.21 U/g Hb), but this was not significant. Activity levels of GSH-PX and GR in diabetics (62.33 ± 36.29 and 7.17 ± 5.51 U/g Hb, respectively) were higher than in controls (24.62 ± 11.2 and 3.16 ± 2.95 U/g Hb, respectively). The statistical difference in enzyme activity of both GSH-Px and GR was significant (*P* <0.05).

**Conclusion:**

The increasing production of free radicals and changes in activity levels of antioxidant enzymes in order to scavenge free radicals and/or the effect of diabetes on the activity levels of antioxidant enzymes has an important effect on diabetic complications and insulin resistance. Evaluation of the levels of antioxidant enzymes and antioxidant factors in patients at different stages of the disease, and pharmaceutical and nutritional interventions, can be helpful in reducing oxidative stress in type 2 diabetic patients. There were positive relationship between BMI and the activity of antioxidant enzymes including SOD, GR and GPX in both groups.

## Introduction

Type 2 diabetes mellitus (DM) is a metabolic disorder that is a major health problem worldwide [1]. The World Health Organization has reported that the global prevalence of diabetes will increase, from 2.8% in 2000 to 4.4% in 2030 [2]. Data from the SURFNCD study in Iran show that 7.7% of Iranian adults (*n* = 2 million) and 16.8% (*n* = 4.4 million) have diabetes and impaired fasting glucose, respectively [3].

Free radicals are reactive molecules produced naturally in the human body during metabolic reactions. High levels of free radicals damage cellular proteins, membrane lipids, and nucleic acids, and eventually lead to cell death. Free radicals play an important role in the pathogenesis of many chronic diseases, including atherosclerosis, myocardial failure, immune diseases, and type 2 diabetes. Free radicals include reactive oxygen species (ROS) and reactive nitrogen species [4]. In healthy subjects, antioxidant compounds counter the effects of free radicals [2]. Antioxidants, which are produced either endogenously or are derived from dietary sources, are categorized into two groups: enzymatic and non-enzymatic. Catalase, superoxide dismutase (SOD), glutathione reductase (GR), glutathione peroxidase, tyrodoxin reductase, ariel esterase, and paraoxonase are included in the enzymatic group, while the non-enzymatic group includes vitamins A, C, and E, carotenoids, glutathione, flavonoids, other compounds such as α-lipoic acid and coenzyme Q10, and copper, zinc, magnesium, and selenium [3]. Oxidative stress is defined as the increased generation of free radicals and/or the impaired compensatory response of endogenous antioxidant defenses, both observed in type 2 diabetes [5]. Oxidative stress is a pathologic condition resulting from either increased production of free radicals or decreased levels of antioxidants. Hyperglycemia, per se or by the promotion of lipid peroxidation of low-density lipoprotein (LDL) can result in the production of free radicals [6]. {Please check the change} Diabetes is a metabolic disorder and is generally accompanied by increased levels of free radicals and decreased concentration or activity of antioxidants. Studies have shown that serum concentrations of SOD and other antioxidants such as vitamin E and α-lipoic acid are decreased in type 2 diabetic patients. There is evidence that deficiency of catalase in erythrocytes is associated with increased risk of diabetes [4]. There is considerable evidence that oxidative stress plays a key role in insulin resistance, impaired insulin secretion and many of the complications of diabetes such as micro-/macro vascular damage [7-10].

There are several mechanisms that increase the intra- and extracellular concentration of glucose resulting in oxidative stress, including the auto-oxidation of glucose [11], the non-enzymatic glycation of proteins, and the increased production of glucose derived from advanced glycosylation end products and activation of the polyol pathway [12]. In this pathway, NADPH, a necessary cofactor for glutathione peroxidase activity, is utilized causing increased concentration of NADH, which is essential for the activation of the enzyme NADH oxidase that causes oxidative stress [13].

There is evidence that hyperglycemia-induced oxidative stress occurs before the onset of diabetic complications. ROS have an important role in the onset and progression of the disease, in addition to that in various physiologic and pathophysiologic complications [14].

The aim of this study was to compare the activities of antioxidant enzymes (SOD, GR, and glutathione peroxidase) and total antioxidant capacity (TAC) in diabetic patients compared with healthy controls.

## Methods

### Participants

In this cross-sectional study 200 subjects (age range 20–80 years; 100 type 2 diabetic patients from the Iranian Diabetes Association and 100 healthy subjects from the staff of Tehran University of Medical Sciences, Tehran, Iran) were selected by random sampling methods. Exclusion criteria included pregnancy, lactation, use of drugs affecting the lipid profile or calcium and bone metabolism, chronic disorders of the liver or kidney, endocrinologic disorders such as hypo- or hyperthyroidism and hyperparathyroidism, smoking, insulin injection, use of anticonvulsive drugs, and vitamin D or calcium supplementation. A written consent form approved by the Ethics Committee of Tehran University of Medical Sciences was signed by each participant.

### Sample collection

After overnight fasting, 10 ml of peripheral blood was withdrawn by a trained nurse. Blood samples were collected in three trace element-free tubes: first tube for serum separation and second tube containing EDTA for plasma separation and third tube used for Hb measurement. Plasma samples were separated from cells by centrifugation at 3000 rpm for 10 min, and the remaining blood was washed three times with 9 g/l NaCl solution. Cell membranes were removed by centrifugation at 1200 rpm for 5 min at 4°C. The hemolysates were then used to determine antioxidant enzyme levels. Serum was separated by centrifugation with coagulated blood at 1000 rpm for 10 min at 4°C. All samples were then stored at −79°C.

Anthropometric data, including weight and height were measured using a Seca scale (Seca 725; GmbH & Co., Hamburg, Germany), with subjects standing wearing light clothes and no shoes. Weight and height were measured to the nearest 0.1 kg and 0.5 cm, respectively. Body mass index (BMI) was defined as weight (kg) divided by height squared (m^2^). Obesity was defined as BMI >30 kg/m^2^. To decrease the seasonal variability in biochemical determinations, our sampling was performed between April and June of 2010.

### Measures

Fasting plasma glucose was measured using glucose-oxidase by a Pars Azmoon kit (Pars Azmoon Co., Tehran, Iran) and plasma insulin by using the radioimmunoassay method (Biosorce kit, Denmark). Glycated hemoglobin (HbA_1_C) concentration was determined using a Nyco Card Reader ІІ analyzer according to the manufacturer’s instructions [15]. A homeostasis model assessment of insulin resistance (HOMA-IR) was calculated using the following formula: $\text{fasting insulin}\;\left(\text{μ}\text{IU}/\text{ml}\right)\times \text{fasting glucose}\;\left(\text{mmol}/\text{l}\right)/22.5$.

Glutathione peroxidase (GSH-Px) activity in erythrocytes was measured spectrophotometrically by a Randox laboratory kit (Ransel, manual) according to the method described by Pagila and Valentin [16]. In this method, GSH-PX enzyme oxidizes glutathione via Cumen hydroperoxidase. In the presence of GR and NADPH, oxidized glutathione is immediately converted to the reduced form, with concomitant oxidation of NADPH to NADP^+^. The decrease in absorbance at 340 nm and 37°C was measured. GR activity in erythrocytes was measured spectrophotometrically with a Randox laboratory kit at 340 nm and 37°C by the method described by Calberg and Mannervik [17]. SOD activity was assayed by the spectrophotometry method of Marklund and Marklund [18]. In this method, the xanthine–xanthine oxidization system {Please check the change} was used as a superoxide radical generator. The absorbance of the reduced product (formazone) was measured at 505 nm, and SOD activity was measured as the degree of inhibition of this reaction. Fasting serum total antioxidant capacity was measured using 2, 2'-azino-bis-3-ethylbenzothiazoline-6-sulfonic acid.

### Statistical analysis

Descriptive statistics were tested before statistical analysis as being normally distributed using histograms and the Kolmogorov–Smirnov method. Variables with normal distribution are expressed as mean ± SD and GR and GSH-PX are expressed as median ± IQR due to lack of normality. Data were analyzed by using Student’s *t*-test, Mann–Whitney U and partial correlation in SPSS software (SPSS version 16.0, Chicago, IL, USA). A value of *P* <0.05 was considered statistically significant.

## Results

One hundred and eighty subjects (35 patients with type 2 diabetes (50 men, 45 women) and 85 healthy subjects (45 men, 40 women)) participated in this study. Baseline demographic and clinical characteristics of the population are shown in Table 1. There were no significant differences with regard to age, weight, and BMI between type 2 diabetic patients and healthy controls. Fasting serum concentrations of calcium were significantly higher in controls than in diabetic patients (Table 1) [3].

**Table 1:** Demographic characteristics of type 2 diabetic patients and healthy subjects

**Type 2 diabetics**	**Controls**	**P**
51.26 ± 11.18	51.55 ± 13.39	0.88
76.96 ± 13.82	73.22 ± 12.98	0.09
26.22 ± 9.30	26.26 ± 4.55	0.98

Data are expressed as Means ± SD.

Fasting blood sugar (FBS), HbA_1_C, and HOMA-IR index were significantly higher in type 2 diabetic patients compared with healthy subjects. However, fasting serum concentrations of insulin showed no significant statistical difference between the two groups (Table 2).

**Table 2:** **Fasting serum concentrations of FBS, HbA**_**1**_**c, insulin, and HOMA-IR in type 2 diabetic patients and healthy subjects**

**Variable**	**Type 2 diabetics**	**Controls**	**P**
**Calcium (mg/dl)**	8.94 ± 0.58	9.13 ± 0.53	0.60
**Phosphorus (mg/dl)**	3.67 ± 0.38	3.69 ± 0.43	0.59
**PTH (pmol/l)**	44.57 ± 16.11	40.94 ± 15.05	0.05
**25(OH) vitamin D (ng/ml)**	20.08 ± 9.30	23.21 ± 11.20	0.04
**FBS (mg/dl)**	174.89 ± 64.18	87.69 ± 9.58	0.001
**HbA**_**1**_**c (%)**	7.54 ± 1.93	4.97 ± 0.55	0.001
**Insulin (μIU/ml)**	11.78 ± 8.90	13.67 ± 8.15	0.13
**HOMA-IR**	89.45 ± 73.91	54.66 ± 31.04	0.001

Data are expressed as Means ± SD.

The activities of GR and GSH-PX were significantly higher in diabetic patients than in healthy controls, although those of SOD and TAC showed no significant difference between the two groups (Table 3).

**Table 3:** Serum concentrations of TAC and activity of antioxidant enzymes (SOD, GR, and GSH-PX) in type 2 diabetic patients and healthy subjects

**Variable**	**Type 2 diabetics**	**Controls**
**TAC (mg/dl)**^**a**^	3.15 ± 1.07	3.22 ± 0.69
**SOD (U/g Hb)**^**a**^	1111.93 ± 354.99	1158.53 ± 381.21
**GR (U/g Hb)**^**b**^	7.17 ± 5.51	3.16 ± 2.95
**GSH-PX (U/g Hb)**^**b**^	62.33 ± 36.29	24.62 ± 11.20

a Mean ± SD.

b Median ± IQR.

* P <0.05.

Table 4 shows the association between the activities of antioxidant enzymes (SOD, GR, and GPX) and serum concentrations of TAC with BMI in type 2 diabetic patients and healthy subjects after adjusted for age, gender, and serum concentrations of FBS, HbA1c, cholesterol, TG, LDL, HDL , PTH and 25(OH)D.

**Table 4:** Partial correlation between activities of antioxidant enzymes (SOD, GR, and GPX), serum levels of TAC and BMI after adjusted for age, gender, and serum concentrations of FBS, HbA1c, cholesterol, TG, LDL, HDL , PTH and 25(OH)D in type 2 diabetic patients and healthy subjects

**Variable**	**Type 2 diabetics**	**Controls**
**SOD**	r = 0.36	r = 0.87
**(U/g Hb)**	P = 0.15	P = 0.02
**GSH-PX**	r = 0.24	r = 0.78
**(U/g Hb)**	P = 0.34	P = 0.05
**GR**	r = 0.15	r = 0.62
**(U/g Hb)**	P = 0.55	P = 0.18
**TAC**	r = − 0.07	r = − 0.40
**(mg/dl)**	P = 0.78	P = 0.42

*P* <0.05 is statistically significant.

## Discussion

According to the results of this study, the mean activity of SOD was higher and the activities of glutathione peroxides and GR lower in type 2 diabetic patients compared with healthy subjects. Pasaoglua and his colleagues performed a study on three groups, including 20 newly diagnosed patients, 20 patients treated with oral antidiabetic agents (OAD), and 20 healthy subjects. Although results showed higher levels of malondialdehyde (MDA) in serum and erythrocytes of diabetics compared with healthy controls, diabetic patients treated with OAD had the highest levels of MDA. In addition, in both diabetic groups SOD activity and concentrations of –SH and GSH were lower and the activity levels of GR, GPX, and lipid per oxidation were higher compared with healthy controls [19].

It was suggested that in the early stage of type 2 diabetes, the antioxidant defense system counters the effects of increased free radicals, but by the advanced stage the balance between generation of free radicals and antioxidant defense is impaired as a result of decreased antioxidant levels or activity.

Likidlilid and his colleagues performed the study to compare glutathione (GSH) level and GPX activity and their relationship with fasting blood sugar (FBS) in type 1 diabetes patients (FPG ≥140 mg/dl) and control group (FPG ≤110 mg/dl). In diabetic patients, there was a negative relationship between FBS and serum levels of GSH, but the same association was not found between FBS and GPX activity [20].

Findings on the effects of diabetes on activity levels of antioxidant enzymes (including SOD, GPX, and GR) in various tissues were contradictory. GPX activity was found to be higher in the liver [21,22], kidney, aorta, pancreas, blood, and erythrocytes and lower in the heart and retina. Animal and human studies have shown different results on the influence of diabetes on SOD activity. Both increase and decrease in SOD activity were reported in erythrocytes, whereas increased activity was seen in plasma and the retina and reduced activity in the pancreas. {Missing citation (work not acknowledged)}

Stefek and colleagues found that SOD activity was increased in diabetic rats after 32 weeks of treatment, but no changes were observed in the aorta. The variation in findings from animal studies may be due to differences in selection of gender, duration of diabetes, tissues investigated, and species of animals used. Likewise, in human studies there are differences with regard to gender, population, duration of diabetes, and levels and types of control of hyperglycemia that may contribute to contradiction in results.

In our study, increased activities of GPX and GR may be a compensatory response to oxidative stress.

Diabetic-induced oxidative stress can promote the development of complications in patients with type 2 diabetes mellitus, and therefore the control of hyperglycemia and oxidative stress must be a key component of treatment.

Aldibasi and his colleagues performed a study on 100 diabetic patients with retinopathy and 60 diabetics without retinopathy as the control group. Data showed that serum levels of hydroxyl 2-deoxy-guanosine (8-OHdG) and MDA were increased and the activity of Cu-Zn SOD was decreased. It is suggested that diabetes-induced oxidative stress plays a key role in the progression of retinopathy in diabetic patients [23].

There is a considerable body of evidence indicating that hyperglycemia may interfere with natural defense of the antioxidant system, in addition to increasing the production of free radicals [24]. Since SOD enzyme is part of the first line of defense against free radicals, it is expected that the activity of this enzyme may be affected by stress oxidative before the other antioxidant enzymes [25]. The data from our study indicate that SOD activity is lower in diabetic patients compared with healthy subjects. There is evidence to show that hyperglycemia is accompanied by the loss of Cu^2+^, which is an essential cofactor in SOD activity, and SOD is inactivated by glycosylation in erythrocytes [25]. Marjani and colleagues conducted a study on 38 type 2 diabetics and 19 healthy subjects and found that serum levels of MDA were higher and SOD activity lower in diabetic patients compared with healthy subjects [26].

It is acknowledged that excess weight and obesity are the main environmental risk factors for chronic diseases such as diabetes. Obesity is a reversible environmental risk factor because it can be both created and reversed by changes in lifestyle [27]. Obesity is an important risk factor for insulin resistance in prediabetic patients and diabetic complications [28]. One possible mechanism for this is the key role of obesity in increasing serum levels of tumor necrosis-α (TNF-α) factor and free fatty acids, which is accompanied by increased oxidative stress. Al-Aubaidy and colleagues found an inverse relationship between serum levels of 8-OHdG (the indicator of DNA destruction in oxidative stress) and obesity [29].

Codoner-Franch carried out research on 20 children with type 1 diabetes and 16 children with obesity, after adjustment for age and gender. Results showed that oxidative stress is observed in both diabetic and obese children, and so it was concluded that there is a synergistic effect between obesity and diabetes that increases oxidative stress [30]. The results from our study showed that there is no statistically significant difference in BMI between diabetics and controls. According to our data, a positive relationship was observed between BMI and the activities of GR, GPX, SOD, in both diabetic patients and controls. These associations were statistically significant between BMI and SOD activity only in healthy subjects.

This study has several limitations, the main one being its cross-sectional nature with no causality effect to report. Variation in the polymorphisms of vitamin D binding protein (DPB) and vitamin D receptor, sunlight exposure, and the effect of vitamin D supplementation on weight gain also need to be considered in the Iranian population. In addition, the activity level of catalase and serum levels of MDA and other vitamins with antioxidant activity were not measured.

## Competing interests

The authors declare that they have no competing interests.

## Authors’ contributions

MDJ, ADJ contributed to the study design and supervised in biochemistry experiments; AS, ET and AMM carried out the experiments and provided the manuscript; MQ involved in the data analysis and the interpretation of results. All authors read and approved the final manuscript.
